# Swedish parents' experiences of their role in treatment for children with congenital limb reduction deficiency: Decision‐making and treatment support

**DOI:** 10.1111/cch.12802

**Published:** 2020-08-18

**Authors:** Lis Sjöberg, Liselotte Hermansson, Helen Lindner, Carin Fredriksson

**Affiliations:** ^1^ School of Health Sciences Örebro University Örebro Sweden; ^2^ Department of Prosthetics and Orthotics, Faculty of Medicine and Health Örebro University Örebro Sweden; ^3^ University Health Care Research Center, Faculty of Medicine and Health Örebro University Örebro Sweden

**Keywords:** family‐centred service, paediatric rehabilitation, parental role, qualitative

## Abstract

**Background:**

Parents of children with congenital limb reduction deficiency have an essential role in making treatment decisions during their child's first years of life. Treatment options usually concern surgical and/or prosthetic treatment. To tailor treatment options to fit different family values and priorities, the family‐centred approach indicates the importance of understanding the parental role in partnership with health care professionals. The aim of this study was to describe parents' experiences of their role in decision‐making and treatment for children with congenital limb reduction deficiency.

**Methods:**

A descriptive design with a qualitative approach was used. Semi‐structured interviews were conducted with 17 parents (12 mothers and 5 fathers) of children with upper and/or lower limb deficiency (mean age 5.9 years). The interview data were analysed using qualitative content analysis with an inductive approach.

**Results:**

Two major themes emerged from the data. The first theme, being a decision maker for someone else, was described as an ambivalent parental role, including collaboration within the family and with health care professionals. The second theme, becoming and being a treatment supporter in the child's everyday life, was made up of four categories: being a supporter of the child in everyday activities, mentoring the child to handle encounters with others, becoming a coordinator of information and being an ‘extended arm’ of the health care provision for the child.

**Conclusions:**

This study enhances our understanding of the parental role in decision‐making and treatment for children with congenital limb reduction deficiency. The results may contribute to the continued development of the family‐centred service approach by providing guidelines for treatment programmes, with the goal of improving decision support and broadening the support for parents during treatment for these children.

**Key messages**

- Health care professionals need to be aware of the differences in parents' abilities and attitudes concerning their role in decision‐making on behalf of their child, in order to respond to the parents' need for support in the decision‐making process.
- Health care professionals also need to develop an understanding for the parents' challenging multiple roles as treatment supporter for the child, regarding encounters with others and day‐to‐day management of the treatment.
- Parents of children with congenital limb reduction deficiency need access to psychosocial support to help them with decision‐making about treatment and during long‐term treatment processes.

## 1 INTRODUCTION

Parents have an essential and challenging role in making both short‐term and long‐term decisions on behalf of their children. It is even more challenging for parents of children with disabilities because they have to make treatment decisions. In the case of parents of a child with congenital limb reduction deficiency (CLRD), the decision‐making process begins when the diagnosis is confirmed, either prenatally or postnatally (Andrikopoulou et al., 2017; Johnson et al., 2016; Lalor, Begley, & Galavan, 2008), and continues throughout the upbringing of the child. The parental role in decision‐making is complex and involves emotional judgements that balance benefits and future needs of the child (Allen, 2014; Bradbury, Kay, Tighe, & Hewison, 1994; Johnson, Johnson, Heyhoe, Fielder, & Dunning, 2018). Health care professionals (HCPs) need to recognize and understand the parental role in decision‐making and subsequent treatment support in order to provide the most appropriate care.

With a prevalence rate of about 4.6 in every 10,000 births (Socialstyrelsen, 2018), CLRD is quite rare but still serious. The deficiency is more common in upper limb than lower limb, or it can be a combination of both upper and lower limb (Ephraim, Dillingham, Sector, Pezzin, & Mackenzie, 2003) and may lead to physical and psychological consequences throughout life if not properly treated (Kaastad, Tveter, Steen, & Holm, 2017; Michielsen, van Wijk, & Ketelaar, 2011; Postema et al., 2016; Varni & Setoguchi, 1992; Watson, 2000; Ylimainen, Nachemson, Sommerstein, Stockselius, & Norling Hermansson, 2010).

With the main goals of improving functionality and appearance, different treatment options are offered to children with CLRD, based on the child's individual need. In Sweden, treatments and assistive devices for children are free of charge. The treatments may include surgical constructions and reconstructions (Lake, 2010; Netscher & Scheker, 1990; Watson, 2000), limb lengthening or shortening procedures (Kaastad et al., 2017), environmental adaptions (Vasluian, van Wijk, Dijkstra, Reinders‐Messelink, & van der Sluis, 2015) such as prescription of prostheses or other assistive devices (Farr, Catena, Martinez‐Alvarez, & Soldado, 2018; Kaastad et al., 2017) or teaching adaptive strategies for daily tasks. Another option to consider is no treatment. In many cases, a combination of treatments requires a multidisciplinary team approach. The professionals most commonly involved as HCPs are physicians, physiotherapists, occupational therapists and prosthetists. For successful outcomes, parental involvement in this team is recommended (Lavigne, Rushton, & Trudelle, 2017; Oliver, Dixon, & Murray, 2020; Postema, van der Donk, van Limbeek, Rijken, & Poelma, 1999). Over the last decade, there has been a shift in health care policies from authoritarian to partnership practice with the aim of strengthening the patient's integrity, participation and autonomy in decision‐making and treatment. *Family‐centred service* is considered to be the best practice in paediatric rehabilitation, incorporating a collaboration in which parents, as experts on their child, work in partnership with HCPs (Johnson et al., 2018; Lavigne et al., 2017; Rosenbaum, King, Law, King, & Evans, 1998). The family‐centred service enhances the ability of HCPs to tailor treatment options to fit different family values and priorities (Law et al., 2005) and to build upon each family's strengths.

Parental involvement has been studied in various paediatric rehabilitation contexts and described from the perspective of the HCPs. Studies conducted with a parental perspective show various factors that influence parental decision‐making regarding their children's treatment (Allen, 2014). The relationship between parents and HCPs (Almasri, An, & Palisano, 2018; McNeilly, Macdonald, & Kelly, 2017), the position or role given to the parents in decision‐making about their children's treatment (Egilson, 2011), social norms and the power of the HCP (Nelson, Caress, Glenny, & Kirk, 2012) were factors that were shown to influence parents in their decision‐making. Clinical experience and earlier research emphasize that parental involvement in treatment is of great importance for the treatment outcomes (D'Arrigo, Copley, Poulsen, & Ziviani, 2019; Durlacher, Verchere, & Zwicker, 2015; Fuller, 1999; Oliver et al., 2020; Setoguchi, 1991; Varni & Setoguchi, 1993; Watson, 2000). Nevertheless, no earlier study has specifically addressed parents' experiences of their role in decision‐making and treatment of their children during the child's first years of life.

In order to strengthen the family‐centred service approach, we need to increase our understanding of the parental role in paediatric rehabilitation for children with CLRD. The aim of this study was to describe parents' experiences of their role in early decision‐making and future treatment support for children with CLRD.

## 2 METHOD

The study had a qualitative design using semi‐structured interviews. The interview transcripts were analysed by qualitative content analysis according to Graneheim and Lundman (2004). The study was approved by the Regional Ethical Review Board in Uppsala, Sweden (approval number: 2016/121/1).

### 2.1 Sample and recruitment

Purposive sampling was used to identify parents from a variety of geographical settings in Sweden, representing different experiences of treatments and encounters with HCPs. Parents were recruited through the Swedish association for children with limb deficiencies and their families, Svensk Dysmeliförening. The inclusion criteria were (i) being a guardian of a child with CLRD, 1–12 years of age; (ii) having contact with an HCP in connection with the child's condition; and (iii) being able to communicate verbally in Swedish. A board member of the association subsequently e‐mailed information and an invitation to participate to parents who fulfilled the inclusion criteria. Parents who were interested in participating in the study responded by e‐mail to the first author (LS), and further information about the study and consent forms were sent to them. To confirm their participation, the parents gave their written consent via e‐mail. The recruitment process was ongoing during data collection, and a total of 17 participants were recruited.

### 2.2 Procedure

All participants were asked to complete a demographic questionnaire about themselves and their child with CLRD. The demographic information is summarized in Tables 1 and 2. For the interviews, a study‐specific interview guide was designed to cover parents' experiences of their roles in decision‐making and treatment for children with CLRD. The questionnaire and the interview guide were tested through discussion in a group of four mothers of children with CLRD. In the questionnaire, one question about siblings was added and a question about describing the limb reduction was reworded to be more understandable for the participants. This test of the interview guide resulted in some of the questions being clarified. The interview guide contained two main questions: (a) *describe your thoughts about the role that you had when you were involved in decision‐making about your child's treatment* and (b) *describe your thoughts about your parental role in a treatment process that goes on for a long time.*

**TABLE 1:** Participants' demographic characteristics

		Participants (*n* = 17)
Parental status	Mother	12
	Father	5
	Biological parent	15
	Adoptive parent^a^	2
Age in years	Median (range)	40 (28–53)
Level of education	Intermediate vocational education	6
	University	11
Family situation	Living with the child, full time	14
	Living with the child, part time	3
	Living with partner	12
	Living without partner	5
Living environment	City/town	10
	Small county/village/suburb	4
	Rural	3
Region of Sweden	North	2
	Middle	10
	South	5

^a^ Child adopted during the first year of life.

**TABLE 2:** Children of participating parents (*n* = 17): Demographic characteristics and treatments

Sex	Girl	9			
	Boy	8			
Age in years	Median (range)	5 (2–12)			
Age at diagnosis	Prenatal	6			
	At birth	9			
	At adoption^a^	2			
Other diagnoses	Allergy	3			
Siblings	Yes	14			
	No	3			
Main daytime location	At home	1			
	Preschool	9			
	School	7			
CLRD condition	Isolated	14			
	Part of a syndrome	3^b^			
CLRD type	Longitudinal	6			
	Transversal	11			
Treatments^c^			Surgery	Prosthesis	Other aids
Affected limb	Upper (unilateral)	12	4	7	3
	Lower (unilateral)	2	2	0	2
	Multiple limbs	3	3	1	3

Abbreviation: CLRD, congenital limb reduction deficiency.

^a^ Child adopted during the first year of life.

^b^ Poland's syndrome *n* = 2, amniotic band syndrome *n* = 1.

^c^ Several treatments possible for the same child.

Interviews were conducted during the period February to June 2017. Each participant made the decision about how the interview would be conducted, face to face (*n* = 3) or by online video telephony (*n* = 14). The interviewer attempted to present questions and discuss issues in a way that encouraged the parents to describe their experiences in their own way. The first author (LS) conducted the interviews, which lasted on average 45 min (range 29–78 min). After the 15th interview, no novel content emerged, and data collection was completed after the 17th interview.

### 2.3 Data analysis

All interviews were audio recorded and transcribed verbatim by an independent research secretary. The transcribed interviews were repeatedly read by the first author to obtain a sense of the whole. The text was analysed using a qualitative content analysis (Graneheim & Lundman, 2004). First, two explicit content areas (Graneheim & Lundman, 2004) were identified in the text: *the role of decision‐making* and *the role of treatment support.* The second step was an inductive analysis in which the text in each content area was divided into meaning units. The meaning units were then condensed, by shortening the text while still preserving the core, and labelled with a code; these codes were abstracted by two of the authors (LS and CF), comparing similarities and differences across codes and sorting them into subcategories. To verify the emerging results, the first author (LS) listened to the interview recordings and re‐read the interview transcripts multiple times.

In the third step, the subcategories were analysed to find similarities and differences and sorted into six categories. Each category was given a name, representing the manifest content. Finally, the underlying meaning, the latent content, of the six categories was formulated into two themes. In order to strengthen credibility and increase trustworthiness of the analysis, the authors (LS, CF and LH) discussed the analysis until consensus about the interpretations was achieved. Data were organized using NVivo 11® qualitative data analysis software.

## 3 RESULTS

Two major themes were identified: *being a decision maker for someone else* and *becoming and being a treatment supporter in the child's everyday life.* The themes, categories and subcategories are presented in Table 3.

**TABLE 3:** Themes, categories and subcategories

Theme 1: Being a decision maker for someone else	
Category	Subcategory
An ambivalent parental role	A self‐evident role—do the best for my child
	A role, maybe not wanted
A collaborative decision maker for someone else	Collaboration within the family
	Collaboration with health care professionals
Theme 2: Becoming and being a treatment supporter in the child's everyday life	
Category	Subcategory
A supporter of the child in everyday activities	Motivating and supporting the child to use assistive devices
	Facilitating and supporting the child's participation in activities
	Supporting the child to see his or her intrinsic value
Mentoring the child to manage encounters with others	Providing the child with tools for handling encounters
	Giving the child the main role in communication with others
A coordinator of information	Sharing information with health care professionals
	Sharing information with the school
An ‘extended arm’—the unexpected role	Performing duties related to aftercare and rehabilitation
	Being an assistant to health care professionals

### 3.1 Being a decision maker for someone else

The first theme is about the parent's role as a decision maker for another person, even when the other person was an infant that they had just started getting to know. Their children had been offered treatment at various clinics in the country, such as hand surgery clinics, orthopaedic clinics or one of the multidisciplinary centres for children with CLRD. Treatments offered were various surgical procedures and prescription of assistive devices (prostheses or orthoses), or a combination of these.

Decisions regarding the child's treatment were mainly made during the child's first years of life, in collaboration with HCPs. The parents took on a role as proxy in decision‐making about early treatment options, with the mission of making the best decisions for their child, both for the present and for the future. Their descriptions of decision‐making included easy decisions, such as minor surgeries or prescription of assistive devices, and difficult decisions, such as major surgeries. Most parents had made a single decision, but some had made repeated decisions related to treatments and complications during the child's upbringing. The parents' experiences of their role in decision‐making for someone else are described in the following categories: *an ambivalent parental role* and *a collaborative decision maker for someone else.*

#### 3.1.1 An ambivalent parental role

Parents experienced their role as decision maker for their child as a part of general parenthood, with their pre‐existing expectations that they would be making decisions leading to the best welfare and opportunities for their child. Most parents expressed willingness to take an essential role in decision‐making for their child and felt entitled to do so. One parent said: ‘We brought him into the world. I wouldn't want anyone else [the HCP] to come in and tell us what they would do’ (P16). To have the role of decision maker and do what is best for the child could also mean postponing some crucial decisions for the child, instead keeping the treatment options open as long as possible, perhaps until the child could make independent decisions. However, part of the experience they described was of not wanting to take on this role as decision maker for somebody else. Having to make early treatment decisions was associated with anxiety and uncertainties. One parent stated, ‘In this case, I would prefer that someone come in and say that this is the best for her from a medical point of view …’ (P8).

#### 3.1.2 A collaborative decision maker for someone else

Being a parent and a decision maker about the child's treatment means being assigned a role as a collaborator: first, a collaboration between parents, living together or not, and second, between the family and the HCP. The family collaboration in decision‐making was described in terms of the parents informing each other, discussing the issue and making the decision together. In some cases, parents complemented each other, in that one parent had given the other one permission to handle discussions with the HCP and the other parent modified and negotiated a decision. In some families, one parent had given the other one permission to make the decision without any collaboration.

The collaboration between parents and the HCP was expressed as a desire for support from the HCP to help the parent make a decision, not to make the decision for them. A supportive collaboration was when the HCPs were honest, sincere, gave time to exchange thoughts and calmed the parent's emotions aroused by the decision situation. A lack of collaboration and support was experienced where the HCPs were unclear regarding factual information, their recommendations and the decision‐making process. In one case, the HCP made strong recommendations in such a way that the parent felt persuaded rather than involved and supported in the decision. The parent reported:

Well, I know that X [the father] and I have discussed the matter after the first time we had been there and at that time they persuaded us not to use a prosthesis, or did not think we should have a prosthesis (P6).

Part of the experience was also that collaboration with the HCP in decision‐making was strongly focused on the surgical methods and assistive devices, with a lack of information about the psychosocial consequences of the treatments. The parents commented that additional help from a psychosocial perspective would be valuable in the early decision‐making process.

### 3.2 Becoming and being a treatment supporter in the child's everyday life

The second theme concerns the parent's emerging role as a treatment supporter in the child's everyday life. Being the treatment supporter means integrating the child's treatment into the everyday life of the family. This role is described in four categories: *a supporter of the child in everyday activities*, *mentoring the child to manage encounters with others*, *a coordinator of information* and *an ‘extended arm’—an unexpected role.*

#### 3.2.1 A supporter of the child in everyday activities

The parental role of supporting the child in everyday activities includes motivating and supporting the child when introducing the use of an assistive device. The role requires creativity, patience and spending time with the child. Initially, parents had followed the instructions and advice from the HCP and later on developed family routines for the child's use of the assistive device. The parents experienced that the responsibility for the child's everyday use of the device rested entirely on them. In order to encourage device use in everyday activities, several parents had even prioritized and participated with the child in treatment camps and group activities arranged by HCPs. However, some parents expressed feelings of guilt for not supporting the child enough.

But I would say that perhaps she doesn't use her prosthesis very actively, and we are willing to admit that sometimes we could have worked harder on that. I mean, maybe we ought to do more. Train more, or like practice doing something and so on (P7).

When the child for some reason failed to use the assistive device, the parent's supportive role was challenged. In this situation, the parents had tried different methods with the aim of resuming the child's use of the device. The parents described this as sometimes successful, sometimes not, with a desire for more support from the HCPs.

Several parents wanted to give their child opportunities to try to perform various activities without any preconceptions of their own or from others that it would be too difficult. Parents often used their own ingenuity, initiative and effort to solve everyday problems that arose during ongoing treatment, such as providing the child with tailor‐made clothing and shoes suitable for a prosthesis or orthosis, as well as making adjustments to allow the child to participate in various activities. In addition to supporting the child practically, many parents provided the child with emotional support in everyday life to make the child feel valuable and strengthen his or her self‐esteem. One parent expressed it in this way:

But he … he should always know that regardless if he fails at something, or cannot do stuff in life, he will always and anyway be good enough the way he is (P11).

#### 3.2.2 Mentoring the child to manage encounters with others

The parents attempted to prevent the child's deficiency from being a barrier in contact with others. As a kind of mentor, the parents prepared their child to manage encounters by not dramatizing them and by creating an understanding for how others might react in situations where the child's CLRD is obvious. Many parents had tried to provide their children with different tools for managing encounters. One example was to encourage the child to initiate contact with other children and to be a sociable person. Another example was to teach the child how to respond to questions or comments from other people in his or her own way, with parental support if problems arose.

But it is also important to make her aware of the fact that people will ask questions, and that she then has been prepared for how to explain why she has got a small hand and that it is not painful … we have equipped her to answer any question that might come up (P4).

As the child got older, some parents had worked strategically to give the child more of the main role in communication with others, in order to strengthen the child's personality and reduce the focus on the disability. Furthermore, most parents had offered the child opportunities to meet other children with CLRD to identify with. However, the parents experienced difficulties and limitations in their ability to support the child fully. Many of them expressed a need for psychosocial support in finding tools or strategies that could prevent or alleviate their child's problems in encounters with other people.

#### 3.2.3 A coordinator of information

Regarding the child's encounters and interactions with others, the parents had even been acting as a coordinator of information in order to increase awareness and understanding of their child's condition and potential to function optimally. Parents of children at preschool and elementary school mentioned that they had distributed and coordinated information with the relevant staff at the child's school, such as information about routines or methods for the use of assistive devices in school activities. Those who received support from HCPs in coordinating information with the school were appreciative, but only a few parents had been offered that support from their HCP.

During ongoing treatment, part of the parent's role was to act as a coordinator of information within the family and between the family and the HCPs. The information being shared concerned everyday strategies at home, treatment outcomes and planning of further treatments and health care visits.

#### 3.2.4 An ‘extended arm’—the unexpected role

The experience of the role as a treatment supporter also involved being an ‘extended arm’ of the health care team. This included duties of aftercare and rehabilitation. Many of these duties were not expressed explicitly by the HCP, which is why it became an unexpected but crucial role for the parents, to ensure optimal treatment outcomes.

Not so much has been said about the requirement on us as parents if she would receive a prosthesis. And I do understand that it put more pressure on us that she should practice, that we ought to practice at home and be of assistance. But far too little is said about that, instead the focus is on what it could mean for her, how it would benefit her … (P8).

Parents of children undergoing prosthetic treatment were expected to observe the fit and function of the prosthesis and contact the prosthetist for a repair or new fitting before the child had grown out of it. This responsibility was expressed as sometimes challenging, due to the need to find time for such monitoring duties, as well as the family's everyday life. Parents of children who had undergone surgical treatment carried out postoperative care and administered analgesic medication. One parent expressed this as:

Well you are supposed to be the parent, but sometimes you feel like a nurse. And you might feel that way even if you really are not. When you sit there and handle the bandaging material and so on. So you … well it almost feels like we are running an orthopedic clinic at home (P17).

## 4 DISCUSSION

This study describes parents' experiences of their role in decision‐making and treatment support for children with CLRD. To our knowledge, this is the first study that explores this role from the parents' own perspective. Important themes that emerged from the interviews are *being a decision maker for someone else* and *becoming and being a treatment supporter in the child's everyday life.* The findings provide insight that goes beyond a general parental role.

Being a parent of a newborn baby with CLRD means participating in health care with a role in decision‐making about the child's treatment. The parents described this as a self‐evident element of parenting, but also described varying degrees of ambivalence about making treatment decisions on someone else's behalf. Some parents had feelings of anxiety and uncertainty that even made them wish to transfer decision‐making to someone else, to the HCP or to the child later in life. Previous research (Allen, 2014; Bradbury et al., 1994) highlights factors that may influence this role, but no study shows this parental ambivalence in being a decision maker for their child in a treatment context. If any ambivalence is noted in one or both parents during the treatment process, HCPs should consider giving appropriate support in order to understand each parent's ability and attitudes regarding making decisions about the child's treatment. According to the framework of family‐centred service, parents should be given the opportunity to decide how involved they want to be in decision‐making and treatment for their child (Lavigne et al., 2017; Law et al., 2005). However, this requires openness regarding the extent of the involvement. The ambivalence expressed by the parents in our study may be because the level of parental involvement was not clarified by the HCP.

Another aspect of decision‐making was that the support parents received was strongly focused on surgical and orthopaedic options, with a lack of support for other aspects of the child's condition and the family's situation. These parental experiences are in line with a previous study (Andrews, Williams, VandeCreek, & Allen, 2009) showing that most families of children with CLRD received support focusing on medical and rehabilitation aspects of the condition but only a few received support focused on emotional and psychosocial aspects. This lack of emotional and psychosocial support may influence the parents' confidence in their role as decision makers for their child and may even impede the partnership and teamwork in decision‐making that is advocated within the family‐centred service approach (Johnson et al., 2016; Law et al., 2005; Rosenbaum et al., 1998). In the interviews, the parents expressed a wish for the HCP to provide decision support, not to make the decision for them. This indicates a need to strengthen the family‐centred service approach by identifying parents' need for support in decision‐making for children with CLRD.

The parental role in becoming and being a treatment supporter has many features that are evident regardless of what kind of treatment the child undergoes. Many children in our study had received treatments tailored to using some type of assistive device in everyday activities. The parents were expected to support the child on a daily basis to use the assistive device, a responsibility that the parents at times had experienced as demanding and difficult to manage. The finding is consistent with Oliver et al. (2020), who recommend a greater focus on the need for support, especially emotional support, to parents of children provided with artificial limbs. Fuller (1999) also emphasized the need for emotional support to parents during the child's surgery process.

Despite ongoing treatment, the parents supported their children's participation in various leisure activities in order to provide developmental opportunities with others. These activities could bring joy but also frustration for the child, feelings that the parents then had to deal with. This is in line with earlier research indicating that parents of children with physical disabilities experience challenges in enhancing children's participation in activities (Lidman, Himmelmann, Gosman‐Hedstrom, & Peny‐Dahlstrand, 2018; Piskur et al., 2012).

In addition to supporting their child practically and psychosocially in their everyday life, the parents performed a number of duties to support the treatment process. Unexpectedly from the parents' point of view, they were given the role of an ‘extended arm’ of the health care team, in which they carried out direct and indirect treatment duties that were crucial to the treatment outcomes. Earlier research has shown the importance of parental involvement and support for the treatment outcomes (Lavigne et al., 2017; Oliver et al., 2020; Postema et al., 1999). A study by Whiting (2014) suggested that these kinds of duties give the parent a range of additional roles and identified elements of role conflict with their ordinary role as a parent to the child. Furthermore, Whiting describes parents' need for complex planning skills and the ability to combine these duties with the coordination of everyday family life. Our findings are consistent with this and highlight the complex nature of the parental role of being a treatment supporter for a child with CLRD.

### 4.1 Methodological considerations

In common with other studies, this study has its strengths and limitations. In order to include participants with experience of health care in different parts of Sweden, we conducted the recruitment in close collaboration with the Swedish association for children with limb deficiency and their families, because a high percentage of Swedish families with children with CLRD are members. The first author (LS) and a board member of the association had continuous contact throughout the recruitment process. This enabled us to recruit participants from different parts of the country with a distribution that well reflects the population. As members of an association, the participants may have been more motivated to participate in studies than other parents, which might have had an impact on the results.

The recruitment process resulted in 17 participants, less than a third of whom were fathers. This skewed distribution is comparable with other interview studies. However, it can be considered a strength that we succeeded in recruiting fathers to a study on the role of parenting. The potential difference between parents' experiences based on their gender is a topic for future research.

Two different methods of performing interviews were used: face to face and online synchronous video calls. According to Janghorban, Latifnejad Roudsari and Taghipour, (2014), online synchronous interviews are comparable with face‐to‐face interviews and enable data collection from geographically dispersed participants, which may have contributed to the wider variety of experiences we gathered. Furthermore, the online interviews allowed us to accommodate to the parents' schedules. In our study, there were no differences between the two interview methods regarding the length or content of the interviews.

There are some potential limitations in the data collection. The questions in the interview guide were focused on the parents' experiences in decision‐making and treatment for their child. Another central part of the collaboration between parents and HCPs, and of great importance for successful rehabilitation, is goal setting (Stefánsdóttir & Thóra Egilson, 2016). No questions about goal setting were included in the interviews, but in hindsight, such questions might have provided valuable information about the parental role in decision‐making and treatment support. Further studies to explore this in the future are needed.

This study contributes to our knowledge about the experiences of the parental role in decision‐making and treatment support. In further research, we need to investigate the parents' experience of the information they receive from HCPs about CLRD and possible treatment options before the decision‐making. There is also a lack of knowledge about the impact of early decisions and treatments in the long‐term perspective. Therefore, further research is needed about how early treatment for children with CLRD is experienced and perceived later in life by the young people themselves.

## 5 IMPLICATIONS FOR CLINICAL PRACTICE

In the development of rehabilitation for children with CLRD, HCPs need to be aware of the differences in feelings and approaches between parents about making treatment decisions for their children, thus enabling the HCP to respond to the parents' support needs during the process. Furthermore, it is important that HCPs develop an understanding for the parents' challenging multiple roles as supporter of the child, including managing the child's encounters with others and taking responsibility for some aspects of the treatment itself. Our findings show that parents of children with CLRD would like access to psychosocial support before decision‐making about proposed treatment and during ongoing treatment.

## 6 CONCLUSION

Parents of children with CLRD experience a demanding parental role in decision‐making and treatment support. The role involves a great responsibility for making the best decisions for their child, both in the present and for the future, and there is a clear need for decision support. Furthermore, being a treatment supporter is challenging because of the many aspects of support and collaboration during ongoing treatment. The role involves supporting the child in everyday life, being a coordinator with HCPs and schools and performing duties related to treatment. The results of this study will inform HCPs about factors to consider regarding collaboration with parents during the treatment of children with CLRD. The results may even contribute to a continued development of the family‐centred service by providing treatment programme guidelines with the goal of improving decision support and broadening the range of support for parents during the treatment of children with CLRD.

## AUTHOR CONTRIBUTIONS

All authors contributed equally to conception and study design. Sjöberg performed acquisition of data. Sjöberg and Fredriksson performed the data analysis, and all authors contributed to further analysis and interpretation of data. All authors have critically reviewed and revised the manuscript and approved the final manuscript as submitted.

## CONFLICTS OF INTRESTS

The authors report no conflicts of interest.

## Data Availability

The data that support the findings of this study are available from the corresponding author upon reasonable request.
